# Factors affecting the migration intention in medical students in Shiraz; south of Iran: a cross sectional study

**DOI:** 10.1186/s12909-025-07700-y

**Published:** 2025-07-24

**Authors:** Fatemeh Parvizi, Alireza Salehi, Atefeh Seghatoleslam, Mohammad Kia, Mohammadmehdi Pope

**Affiliations:** 1 Department of MPH, School of Medicine, Shiraz University of Medical Sciences, Shiraz, Iran; 2 Department of Biochemistry, School of Medicine, Shiraz University of Medical Science, Shiraz, Iran

**Keywords:** Medical students, Emigration, Iran, Shiraz university of medical sciences

## Abstract

**Background:**

The increasing emigration of human resources, particularly healthcare workers, poses a significant challenge to achieving the sustainable development goal of equitable healthcare access. This study aimed to assess the migration intentions among medical students at Shiraz University of Medical Sciences and to identify the factors that drive or hinder their propensity to emigrate.

**Methods:**

This cross-sectional study employed stratified random sampling. Data were collected anonymously through a researcher-designed questionnaire completed by 403 medical students. The questionnaire’s validity and reliability were established within this study. It comprised three sections: demographics, quantitative and qualitative questions regarding migration intentions, and factors influencing these intentions. Data analysis included bivariate and multivariate methods, with linear regression applied to identify significant predictors.

**Results:**

Among the participants, 70.7% expressed an intention to emigrate, with an average migration propensity score of 5.70 ± 2.32 (out of 10). Significant positive associations were found between migration intentions and variables such as pre-university education region, father’s education level, prior international travel experience, presence of relatives abroad (including degree of kinship), English language proficiency, possession of foreign language certificates, knowledge of additional languages, number of published papers, online communication with individuals living abroad, and access to migration information sources. Multivariate linear regression highlighted privileged pre-university education regions, having close relatives abroad, English language skills, and access to migration information as significant predictors. Among the five categories of migration drivers (personal, economic, social, political, and structural), personal factors—including aspirations for a better life, gaining experience, family welfare, work-life balance, and family satisfaction—were the predominant motivators (29.8%). Conversely, personal barriers such as family dependence, feelings of alienation, language difficulties, and family dissatisfaction were the main obstacles (42.7%).

**Conclusion:**

Enhancing overall welfare, improving work-life balance, increasing salaries, promoting physicians’ social dignity, and strengthening job security are essential strategies to reduce the intention to emigrate and retain skilled healthcare professionals.

**Supplementary Information:**

The online version contains supplementary material available at 10.1186/s12909-025-07700-y.

## Background

Human emigration refers to the movement of individuals from one location to another with the intention of settling, either temporarily or permanently, in a new geographical area [1]. Among different forms of emigration, academic emigration involves the cross-border mobility of professors, researchers, and postdoctoral fellows who seek academic work or career development opportunities abroad [2]. This phenomenon poses a significant global challenge, as it results in the transfer of skilled human resources from countries that need their expertise to other nations where they often settle permanently or semi-permanently [3].

Skilled human resources are a crucial factor of production, and the emigration of elites can substantially impact the development of their countries of origin [4, 5]. Economically, attracting a highly educated individual with a Master’s or doctoral degree from a developing country can be worth up to a million dollars to the destination country. Despite comprising approximately 80% of the global population, developing countries receive only 20% of the world’s income and possess merely 1% of the scientific human resources [6]. This imbalance creates significant challenges for the countries experiencing the brain drain. For example, the cost of training a general practitioner has been estimated at approximately $57,000; if this physician emigrates and works abroad for 30 years, the financial loss to the country of origin can reach $450,000 [7]. Therefore, sound policies are necessary to ensure equitable distribution of healthcare human resources and to support health equity, a key principle of sustainable development [8].

The emigration of medical personnel has led to numerous adverse outcomes, including reduced quality and accessibility of healthcare, workforce shortages, disruptions in healthcare delivery, increased burnout among remaining staff, higher patient wait times, elevated healthcare costs, and the loss of experienced educators and active healthcare professionals [9–12].

International migration of healthcare workers has steadily increased in recent years [13]. Between 2000 and 2010, approximately 1.493 million Iranians emigrated [14]. According to OECD data, about 5,888 Iranian graduates emigrated to OECD countries in 2018, representing an 89% increase since 2000 [15]. The World Health Organization reported in 2017 that Iran ranked seventh worldwide in the number of physician migrants, with approximately 13,000 Iranian physicians relocating to 35 OECD countries—a 40% increase since 2000 [16].

A cross-sectional study by Ahmadi et al. on medical students at Tehran University of Medical Sciences found an emigration intention rate of 61.3%. Factors positively associated with emigration propensity included female gender, elite status, higher socioeconomic status, prior exposure to foreign countries, foreign language proficiency, and research involvement [17]. Similarly, Asadi et al. (2016) reported an emigration intention rate of 54.77% among human resources at the same university. Their study identified older age, longer work experience, unemployment, being single, improved foreign language skills, having relatives abroad, and previous international exposure as significant correlates [18].

Despite the critical importance of academic emigration, limited research has examined the emigration tendencies of Iranian medical professionals. Given that accurate data is essential for identifying emigration patterns and informing future policies, this study aimed to assess the emigration intentions among medical students at Shiraz University of Medical Sciences and to explore the factors influencing these intentions.

## Method

### Participants

This cross-sectional questionnaire-based study was conducted in 2024 among 403 Iranian medical students at Shiraz University of Medical Sciences. Inclusion criteria included being an enrolled student and willingness to participate, while exclusion criteria comprised refusal to participate, being a guest or international student, and having been enrolled for less than three months. Based on prior studies reporting an emigration propensity of 61.3%, the minimum required sample size was calculated to be approximately 372 participants, with a 5% standard deviation (SD) and a 5% alpha error.

The professional doctorate program at Shiraz University of Medical Sciences spans seven years and consists of three stages: basic sciences, pre-clinical, and clinical. The study population was stratified accordingly into these three groups. Using stratified random sampling, the minimum sample size was proportionally allocated to each stratum. After obtaining oral informed consent, self-administered paper questionnaires were distributed during scheduled classes and clinical rotations, ensuring the presence of all students within each educational stage. Measures were taken to protect participant privacy.

The study protocol was approved by the Ethics Committee of Shiraz University of Medical Sciences (Reference number: IR.SUMS.MED.REC.1403.378). Participation was entirely voluntary, with oral informed consent obtained prior to data collection. All collected data were kept confidential by the research team.

### Tools

An extensive literature review identified 80 potential factors influencing emigration propensity, which were incorporated into a researcher-designed questionnaire. Content validity was assessed using the Content Validity Ratio (CVR) and Content Validity Index (CVI) [19]. Fourteen experts in medicine and sociology evaluated each item on a 3-point scale (“unessential,” “useful but not essential,” “essential”). According to Lawshe’s table, items with a CVR below 0.51 were removed. Experts further rated item relevance on a 4-point scale, and items scoring below 0.78 on the CVI were excluded. After these validations, 32 items remained.

Face validity was tested through a pilot study involving 30 medical students, with revisions made to enhance clarity based on feedback. The questionnaire demonstrated acceptable internal consistency, with a Cronbach’s alpha of 0.85.

The questionnaire comprised three sections: demographic information (e.g., age, gender, marital status, pre-university education region, educational stage, parents’ education levels, family emigration history, foreign language proficiency, publication record, membership in the Iranian National Elite Foundation (INEF), economic and social status, residence, and social media connections abroad); qualitative yes/no questions on emigration intention; and quantitative assessment of emigration propensity using a 0–10 scale. Additionally, factors driving or hindering emigration were explored (see Table 1). The questionnaire was administered in Persian, and all responses were anonymous.

**Table 1:** Drivers and barrier to emigration propensity

Potential Drivers and Barriers	Number (percentage%)	Examples of drivers & Barriers
Personal factors		Achieving a better life, gaining more experiences, providing family welfare, balancing between work and leisure hours, family desire
Yes	248 (61.50)	
No	154 (38.20)	
Occupational factors		Discrimination between disciplines, concerns about the future of employment (job security), problems in the work environment and unhealthy professional relationships, overwhelming workload, difficulty in career promotion.
Yes	208 (51.60)	
No	194 (48.10)	
Economic factors		Achieving more job rights and facilities, insufficient job support
Yes	238 (59.10)	
No	164 (40.70)	
Structural factors		The problems of the educational system and the health system, the problems of the research field, the allocation of unfair quotas, exhaustion and technological backwardness, the low quality of education, the invalidity of the university degrees
Yes	198 (49.10)	
No	204 (50.60)	
Sociopolitical factors		Political-social restrictions inside the country, the recent decrease in the social status of physicians in the society
Yes	204 (50.60)	
No	198 (49.10)	
Economic barriers		Heavy costs of emigration and living abroad, rising exchange rates
Yes	189 (46.90)	
No	212 (52.60)	
Cultural/ religious barriers		Cultural and religious differences, patriotism and interest in serving in the hometown, communication problems, job discrimination in the destination country
Yes	73 (18.10)	
No	329 (81.60)	
Political barriers		Hard acceptance of students from embargoed countries, military service (military or project), the cost of releasing degrees, hard acceptance of degrees obtained in third world universities.
Yes	121 (30.00)	
No	281 (69.70)	
Personal barriers		Dependence on the family, feeling of alienation, language problems, family dissatisfaction
Yes	225 (55.80)	
No	177 (43.90)	
Occupational barriers		The difference between clinical work and practice in the destination country, lack of familiarity with the work environment, gaps in knowledge and skills, lack of study or work opportunities abroad, difficulty in preparing for emigration, employment in a position lower than capabilities, the requirement to pass professional courses that have already been completed
Yes	104 (25.80)	
No	298 (73.90)	

In the Iranian pre-university education system, students are categorized into regions based on access to educational resources: Region 1 denotes the most privileged, Region 3 the least privileged, and Region 2 is intermediate. Some students belong to special groups with specific privileges (e.g., children of military personnel or university faculty) and are compared within their group.

### Analysis

Data analysis was performed using IBM SPSS Statistics version 26. Quantitative variables were summarized using means, standard deviations, minima, and maxima, while qualitative variables were presented as frequencies and percentages. Statistical tests employed included t-tests, ANOVA, Pearson correlation, Chi-square, and linear regression. Normality of data distribution was confirmed. A *p*-value of less than 0.05 was considered statistically significant.

## Results

A total of 403 medical students participated in the study, with a mean age of 22.84 ± 2.32 years. Approximately half of the participants were male (50.9%) and the majority were single (91.6%). Of the participants, 79 (19.6%) were in the basic sciences phase, 47 (11.7%) in the pre-clinical stage, and 277 (68.8%) in the clinical phase.

Among the 403 students, 285 (70.7%) expressed an intention to emigrate. The mean emigration propensity score was 5.70 ± 2.32 on a scale of 0 to 10. Demographic characteristics and their correlations with emigration propensity, along with corresponding *p*-values, are summarized in Table 2.

**Table 2:** Demographic characteristics and emigration propensity

Demographic Characteristics	Number (percentage%)	Emigration propensity Mean(SD)	*P*. value
Gender			0.891^a^
Male	198 (49.10)	5.72 (3.27)	
Female	205 (50.90)	5.67 (3.65)	
Marital status			0.285^a^
Single	369 (91.60)	5.75 (3.42)	
Married	34 (8.40)	5.08 (3.92)	
Educational phase			0.315 ^b^
Basic	79 (19.60)	5.17 (3.48)	
Preclinical	47 (11.70)	5.97 (2.98)	
Clinical	277 (68.8)	5.80 (3.53)	
Pre-university education region			0.002* ^b^
One	184 (45.70)	6.30 (3.34)	
Two	102 (25.30)	5.62 (3.56)	
Three	100 (24.80)	4.87 (3.36)	
Other	15 (3.70)	3.9 (3.63)	
Father’s educational attainment			0.001* ^b^
Diploma and less	95 (26.60)	4.93 (3.39)	
Master and bachelor	144 (35.70)	5.35 (3.51)	
Doctorate and higher	164 (40.70)	6.42 (3.34)	
Mother’s educational attainment			0.061 ^b^
Diploma and less	135 (33.50)	5.18 (3.46)	
Master and bachelor	157 (39.00)	5.76 (3.27)	
Doctorate and higher	111 (27.50)	6.22 (3.68)	
parents’ migration for education			0.112 ^a^
Yes	33 (8.20)	6.63 (3.46)	
No	370 (91.80)	5.61 (3.46)	
Traveling abroad experience			0.003* ^a^
Yes	217 (53.8)	6.16 (3.46)	
No	186 (46.20)	5.15 (3.40)	
Residence abroad experience			< 0.0001* ^a^
Yes	37 (9.20)	7.60 (2.97)	
No	366 (90.80)	5.50 (3.46)	
Having relatives living abroad			0.003* ^a^
Yes	324 (80.40)	5.94 (3.45)	
No	79 (19.60)	4.64 (3.35)	
Degrees of relative consanguinity			0.001* ^b^
One	82 (20.30)	6.83 (3.38)	
Two	153 (38.00)	6.07 (3.37)	
Three or more	91 (22.60)	4.90 (3.25)	
English language skills			< 0.0001* ^b^
Weak	24 (6.00)	3.95 (3.15)	
Intermediate	269 (66.70)	5.35 (3.43)	
Advanced	110 (27.30)	6.88 (3.29)	
Having language certificate			0.045* ^a^
Yes	67 (16.60)	6.48 (3.13)	
No	336 (83.40)	5.54 (3.51)	
Knowing a language other than English			0.006* ^a^
No	291 (72.20)	5.39 (3.37)	
Yes	112 (27.80)	6.48 (3.60)	
Having published papers			0.062 ^b^
None	339 (84.10)	5.56 (3.46)	
One or two	48 (11.90)	6.02 (3.28)	
More than two	16 (4.00)	7.56 (3.74)	
Articles published in scientific databases*			0.029* ^b^
None	349 (86.60)	5.50 (3.48)	
One or two	40 (9.90)	6.72 (2.82)	
More than two	13 (3.20)	7.23 (4.04)	
Membership of Iranian National Elite Foundation			0.839 ^a^
Yes	92 (22.80)	5.62 (3.64)	
No	311 (77.20)	5.71 (3.42)	
Economic status			0.077 ^b^
Less than mean	36 (8.90)	4.78 (3.82)	
Mean	191 (47.40)	5.51 (3.37)	
More than mean	178 (43.50)	6.09 (3.45)	
personal income			0.245 ^a^
Yes	100 (24.80)	5.33 (3.63)	
No	303 (75.20)	5.81 (3.41)	
Self-perception of one’s social position			0.639 ^b^
Less than mean	16 (3.90)	6.13 (3.94)	
Mean	41 (10.20)	5.19 (3.40)	
More than mean	57 (14.20)	5.73 (3.75)	
Being Native			0.023* ^a^
Native	229 (56.80)	6.36 (3.51)	
Not native	174 (43.20)	5.24 (3.36)	
Residence			< 0.0001* ^b^
Dormitory	131 (32.50)	4.81 (3.30)	
Living at home with parents	218 (45.10)	5.94 (3.52)	
Living at home without parents	54 (13.40)	6.86 (3.16)	
Social media activity			0.102 ^b^
Less than mean	57 (14.10)	4.81 (3.75)	
Mean	185 (45.90)	5.71 (3.48)	
More than mean	161 (40.00)	5.97 (3.32)	
The number of people living abroad whom one is connected with via social media			< 0.0001* ^b^
None	166 (41.20)	5.14 (3.47)	
One or two	138 (34.20)	5.48 (3.41)	
More than two	99 (24.60)	6.91 (3.26)	
Access to emigration information sources			< 0.0001* ^b^
None	76 (18.90)	4.19 (3.45)	
One or two	159 (39.50)	5.51 (3.47)	
More than two	168 (41.70)	6.53 (3.23)	

*Scientific data base including PubMed, Scopus, web of science, ^a^ T-test, ^b^ ANOVA

Regarding pre-university education regions, students from Region 1 (the most privileged area) demonstrated a significantly higher inclination to emigrate compared to those from other regions (*p* = 0.002). This association remained significant in multivariate analysis (*p* = 0.014). Detailed results of the multivariate analysis are presented in Table 3.

**Table 3:** Multivariate analysis of variables influencing emigration propensity

Significant Variables from Bivariate Analysis	B	t	*P*.value
Pre-university education region	-0.161	− 0.2.463	0.014*^a^
Father’s educational attainment	0.024	0.420	0.675 ^a^
Traveling abroad experience	0.051	0.871	0.384 ^a^
Residence abroad experience	-0.064	-1.113	0.267 ^a^
Having relatives living abroad	-0.004	-0.064	0.949 ^a^
Degrees of relative consanguinity	-0.122	-2.100	0.037* ^a^
English language skills	0.147	2.380	0.018* ^a^
Having language certificate	0.015	0.252	0.801 ^a^
Knowing a language other than English	0.079	1.442	0.150 ^a^
Having published papers	0.061	1.130	0.259 ^a^
Being Native	0.088	0.859	0.391 ^a^
Residence	0.011	0.104	0.917 ^a^
Connection with people living abroad on social media	-0.031	-0.506	0.613 ^a^
Access to emigration information sources	0.159	2.722	0.007* ^a^

^a^ Linear regression

**Fig. 1:**
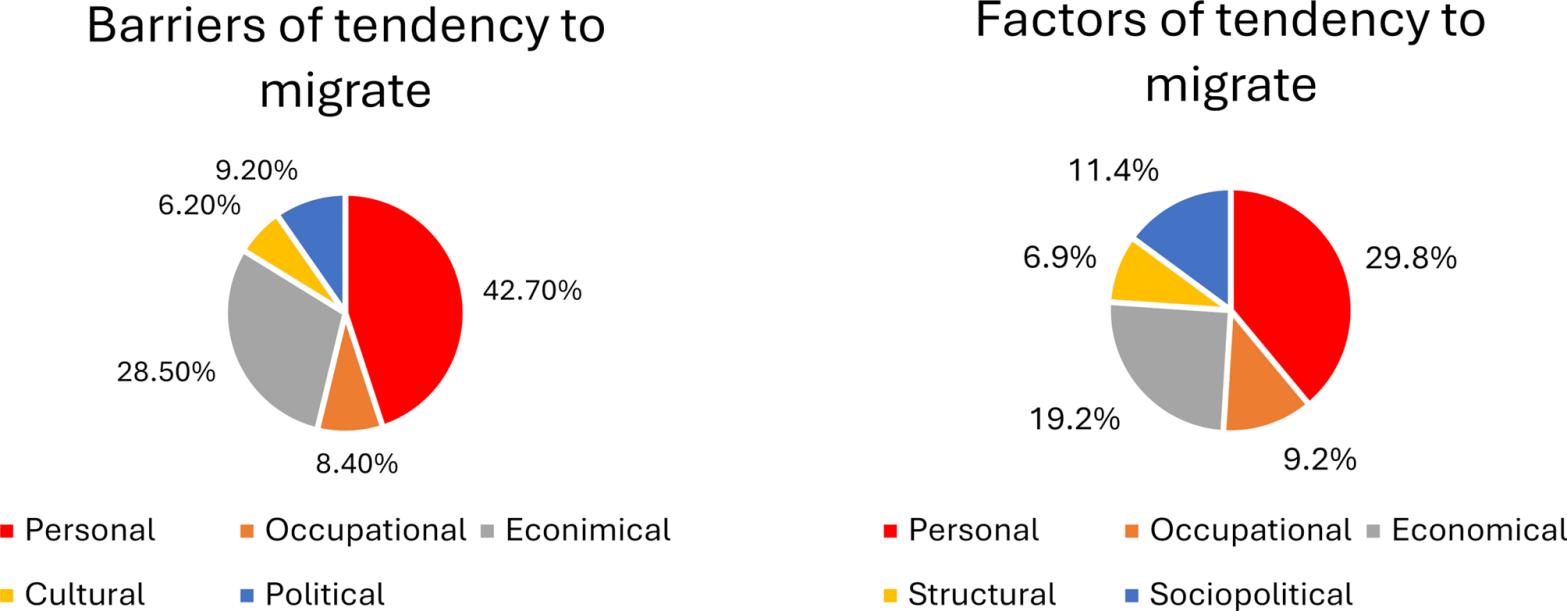
Drivers and barriers to emigration propensity

Participants were also surveyed about the factors influencing their emigration intentions. As presented in Table 1, drivers of emigration propensity were grouped into five categories: personal, occupational, economic, structural, and socio-political factors. Similarly, barriers to emigration were categorized into economic, cultural-religious, political, personal, and occupational factors. Notably, personal factor was identified as the most significant factor, contributing to 29.8% of the drivers and 42.7% of the barriers to emigration propensity (Fig. 1).

## Discussion

This study found that 70.7% of Shiraz University of Medical Sciences students expressed a tendency to emigrate, with an average emigration propensity score of 5.7 out of 10. This rate is higher than that reported in similar Iranian studies, such as Asadi et al. (60.9%) [18] and Ahmadi et al. (61.3%) [17], and exceeds emigration tendencies reported among medical students in Pakistan (50.6%) [20], Egypt (49.1%) [21] Ethiopia (53%) [22], Lithuania (39%) [24], and some European countries like Romania (85%) [23]. These findings suggest a rising trend of emigration propensity in Iran, as well as a comparatively high rate relative to other countries.

While this study did not find significant correlations between emigration tendency and gender or age, previous research offers mixed results. Ahmadi [17] reported higher emigration propensity among males, whereas studies in China [25] and Jordan [26] found females or older individuals more inclined to emigrate. Similarly, Asadi et al. [18] observed younger individuals tending to emigrate more, contrasting with findings from Jordan and Greece [26, 27]. The lack of association in the current study may reflect the relatively homogenous age range of participants or differing cultural factors.

The impact of education level on emigration varies internationally. Studies from Ireland and Ethiopia show higher emigration tendencies among well-educated individuals [22, 28], while Ghanaian students with lower educational levels reported greater propensity [21]. Consistent with other Iranian studies [17, 18, 29], no significant link was found between educational stage or marital status and emigration tendency in this sample, possibly due to the low proportion of married participants.

Consistent with prior research [17, 18, 24, 26], this study found that experiences such as traveling abroad, residing overseas, having relatives abroad, social media connections with expatriates, and access to emigration information significantly increased emigration propensity. These findings support the concept that prior exposure to foreign countries and social networks abroad may both attract and facilitate emigration.

Foreign language proficiency, including English and other languages, was also positively associated with emigration propensity, echoing previous findings [17, 18]. This relationship may be bidirectional, as those interested in emigrating are more likely to acquire language skills, while language proficiency also facilitates successful migration.

Socioeconomic factors presented a complex picture. While economic status and personal income did not significantly impact emigration tendency, students from more privileged pre-university education regions and those with fathers holding higher education degrees were more likely to consider emigration. These findings align with studies in Iran and Jordan demonstrating greater emigration propensity among individuals from urban and higher socio-educational backgrounds [17, 26]. Parents’ educational attainment may foster academic ambition and resources conducive to migration.

Academic productivity, measured by the number of published papers, was also positively correlated with emigration tendency, consistent with findings from Iran and Jordan [17, 26]. This may reflect the desire of academically active students to seek advanced training and career opportunities abroad.

Personal factors emerged as the primary drivers and barriers to emigration propensity in this study. Similar findings were reported by Asadi et al., who identified personal aspirations such as achieving a better life as the leading motivator [18]. Other studies highlight political [30], economic, and educational factors as significant motivators for migration [26–28]. Across diverse contexts, the pursuit of improved quality of life remains a predominant factor influencing medical students’ emigration intentions.

### Limitations

This study’s cross-sectional design limits causal inferences, and the findings may reflect the socio-political context at the time of data collection. However, its strengths include an adequate sample size and inclusion of students across different educational stages.

### Recommendations

Further longitudinal and qualitative research is needed to explore the underlying reasons for emigration propensity and to inform effective policy interventions aimed at retaining skilled medical personnel.

## Conclusion

This study demonstrated that medical students with higher education levels and socio-economic status exhibit a greater tendency to emigrate. The findings provide valuable insights for health managers and policymakers regarding the key drivers and barriers influencing students’ emigration intentions. Strategies aimed at improving general welfare, enhancing work-life balance, increasing salaries, elevating the social status and dignity of physicians, and strengthening job security may effectively reduce emigration propensity. Addressing these underlying factors is essential to retain specialized and skilled medical professionals and mitigate physician emigration in the future.

## Electronic supplementary material

Below is the link to the electronic supplementary material.

Supplementary Material 1

## Data Availability

The datasets used and analyzed during the present study are available from the corresponding author on reasonable request.

## Declarations

### Authors’ information

AS is an epidemiologic professor at Shiraz University of Medical Sciences.

FP is a medical student at Shiraz University of Medical Science.

MK is a medical student at Shiraz University of Medical Science.

AS is a biochemistry professor at Shiraz University of Medical Sciences.

MMD is a medical student at Shiraz University of Medical Science.

## Abbreviations

SUMS: Shiraz University of Medical Science
OECD: Organization for Economic Co-operation and Development
SD: Standard Deviation
N: Number
