# Supplementary material for: Factors affecting the migration intention in medical students in Shiraz; south of Iran: a cross sectional study
Source: BMC Med Educ. 2025 Jul 24;25:1108. doi: 10.1186/s12909-025-07700-y (PMC12288287; doi:10.1186/s12909-025-07700-y)
Supplement: Supplementary file 1 — Supplementary Material 1 [file 12909_2025_7700_MOESM1_ESM.pdf]

Dear Student,

The following questionnaire has been prepared to investigate the willingness of medical students to migrate and the factors affecting it. Please help us in conducting this study by spending a few minutes of your time and providing accurate and accurate answers. This questionnaire is anonymous and nameless.

1.Gender:

Male

Female

2.Age:

3.Marital status:

Single

Married

4.Educational phase:

Basic

Preclinical

Clinical

5.Pre-university education region:

One

Two

Three

Other

6.Father’s educational attainment:

Diploma and less

Master and bachelor

Doctorate and higher

7.Mother’s educational attainment:

Diploma and less

Master and bachelor

Doctorate and higher

8.parents’ migration for education:

Yes

No

9.Traveling abroad experience:

Yes

No

10.Residence abroad experience:

Yes

No

11.Having relatives living abroad:

Yes

No

12.Degrees of relative consanguinity:

One

Two

Three or more

13.English language skills:

Weak

Intermediate

Advanced

14.Having language certificate:

Yes

No

15.Knowing a language other than English:

No

Yes

16.Having published papers:

None

One or two

More than two

17.Articles published in scientific databases*:

None

One or two

More than two

18.Membership of Iranian National Elite Foundation:

Yes

No

19.Economic status:

Less than mean

Mean

More than mean

20.personal income:

Yes

No

21.Self-perception of one’s social position:

Less than mean

Mean

More than mean

22.Being Native:

Native

Not native

23.Residence:

Dormitory

Living at home with parents

Living at home without parents

24.Social media activity:

Less than mean

Mean

More than mean

25.The number of people living abroad whom one is connected with via social media

None

One or two

More than two

26.Access to emigration information sources:

None

One or two

More than two

27. Do you have any desire to immigrate?

Yes

No

28. How would you rate your willingness to migrate from zero to ten?

| Variables | drivers & Barriers |
|---|---|
| **Personal factors** | Achieving a better life, gaining more experiences, providing family welfare, balancing between work and leisure hours, family desire |
| **Occupational factors** | Discrimination between disciplines, concerns about the future of employment (job security), problems in the work environment and unhealthy professional relationships, overwhelming workload, difficulty in career promotion. |
| **Economic factors** | Achieving more job rights and facilities, insufficient job support |
| **Structural factors** | The problems of the educational system and the health system, the problems of the research field, the allocation of unfair quotas, exhaustion and technological backwardness, the low quality of education, the invalidity of the university degrees |
| **Sociopolitical factors** | Political-social restrictions inside the country, the recent decrease in the social status of physicians in the society |
| **Economic barriers** | Heavy costs of emigration and living abroad, rising exchange rates |
| **Cultural/ religious barriers** | Cultural and religious differences, patriotism and interest in serving in the hometown, communication problems, job discrimination in the destination country |
| **Political barriers** | Hard acceptance of students from embargoed countries, military service (military or project), the cost of releasing degrees, hard acceptance of degrees obtained in third world universities. |
| **Personal barriers** | Dependence on the family, feeling of alienation, language problems, family dissatisfaction |
| **Occupational barriers** | The difference between clinical work and practice in the destination country, lack of familiarity with the work environment, gaps in knowledge and skills, lack of study or work opportunities abroad, difficulty in preparing for emigration, employment in a position lower than capabilities, the requirement to pass professional courses that have already been completed |

29. Which of the following categories do your reasons for immigrating fall into? (You can select multiple items)

Personal

Occupational

Economical

Structural

Socio-political

30. What is the main reason for your desire to migrate?

Personal

Occupational

Economical

Structural

Socio-political

31. Which of the following categories do your barriers to immigration fall into? (You can select multiple items)

Personal

Occupational

Economical

Political

Religious-cultural

32. What is the main obstacle to your desire to migrate?

Personal

Occupational

Economical

Political

Religious-cultural

Thank you for your attention.
